# Human cytomegalovirus and Epstein-Barr virus infections, risk factors, and their influence on the liver function of patients with acute-on-chronic liver failure

**DOI:** 10.1186/s12879-018-3488-8

**Published:** 2018-11-16

**Authors:** Jianhua Hu, Hong Zhao, Danfeng Lou, Hainv Gao, Meifang Yang, Xuan Zhang, Hongyu Jia, Lanjuan Li

**Affiliations:** 1 State Key Laboratory for Diagnosis and Treatment of Infectious Diseases, Collaborative Innovation Center for Diagnosis and Treatment of Infectious Diseases, The First Affiliated Hospital, College of Medicine, Zhejiang University, 79 QingChun Road, Hangzhou, 310003 Zhejiang China; 2 Shulan (Hangzhou) Hospital, 848 Dongxin Road, Hangzhou, 310004 Zhejiang China

**Keywords:** Acute-on-chronic liver failure, Human cytomegalovirus, Epstein-Barr virus, Hepatitis B virus

## Abstract

**Background:**

Studies on human cytomegalovirus (HCMV) and Epstein-Barr virus (EBV) have focused primarily on the immunosuppressed population. Few studies have considered immunocompetent and not severely immunocompromised patients. We determined the infection rates of HCMV and EBV, their risk factors and their influence on liver function in patients with HBV-related acute-on-chronic liver failure (ACLF).

**Methods:**

Patients infected with ACLF-based hepatitis B virus (HBV) from 1 December 2016 to 31 May 2018 were enrolled in our study and were divided into infected and uninfected groups. The risk factors for HCMV and EBV infection and their influence on liver function were analysed.

**Results:**

A total of 100 hospitalized patients with ACLF due to HBV infection were enrolled in this study. Of these patients, 5% presented HCMV deoxyribonucleic acid (DNA) and 23.0% presented EBV DNA. An HBV DNA count of < 1000 IU/mL increased the occurrence of HCMV infection (*P* = 0.003). Age, especially older than 60 years, was a risk factor for EBV infection (*P* = 0.034, *P* = 0.033). HCMV-infected patients had lower alanine aminotransferase (ALT) levels; albumin levels and Child–Pugh scores in EBV-infected patients were higher than those in uninfected patients.

**Conclusions:**

HCMV and EBV were detected in patients with ACLF caused by HBV infection. Lower replication of HBV (HBV DNA < 1000 IU/mL) may increase the probability of HCMV infection; age, especially older than 60 years of age, was a risk factor for EBV infection. HCMV infection may inhibit HBV proliferation and did not increase liver injury, while co-infection with EBV may influence liver function and may result in a poor prognosis.

## Background

Human cytomegalovirus (HCMV) is a β human herpesvirus with positive rates of antibodies of 50–100% [1, 2]. Primary HCMV infection is often acquired during early childhood, and in later development stages, HCMV establishes lifelong latency or persistence within a person through cells of myeloid lineage [1] or granulocyte-monocyte lineage [3]. However, reactivated or exogenous reinfection with HCMV may occur and may become a major viral cause of morbidity and mortality in immunocompromised patients, such as organ or haematopoietic stem cell transplant (HSCT) recipients [4–6] and patients with acquired immune deficiency syndrome (AIDS) [1, 2].

Epstein-Barr virus (EBV) is a γ human herpesvirus with positive rates of antibodies of up to 90% [7, 8]. Primary EBV infection mostly manifests as infectious mononucleosis, with fever, angina, lymphadenopathy, and liver and spleen enlargement. After a primary infection occurs, EBV also develops a lifelong latency in B cells [9]. Latent EBV may be reactivated under certain conditions, thereby inducing abnormal B cell proliferation and leading to neoplastic disease.

Studies on HCMV have focused mostly on immunosuppressed individuals [2, 4, 5]. Studies on EBV have mostly examined patients with neoplastic disease [10] and post-transplant lymphoproliferative disorder following HSCT [5, 11, 12]. Limited studies have considered immunocompetent and not severely immunocompromised patients.

Acute liver failure (ALF) is a well-established medical emergency defined as a severe liver injury. However, a proportion of patients who present features mimicking ALF suffer from an underlying chronic liver disease or liver cirrhosis. These patients, who have been defined as patients with acute-on-chronic liver failure (ACLF), display various degrees of immune disorders and short-term mortality rates [13]. HCMV may induce fulminant hepatic failure [14, 15], and EBV may trigger acute liver failure [15–17].

HCMV may be reactivated in patients with cirrhosis [18–20]. Rosi, S. et al. [19] recently revealed that HCMV causes or contributes to hepatic decompensation or ACLF in patients with cirrhosis even if they are not severely immunocompromised. However, the patients in the above study [19] had alcohol-related and mixed-aetiology cirrhosis (hepatitis C virus and alcohol-related cirrhosis). To date, studies have yet to describe HCMV or EBV infections in patients with ACLF due to hepatitis B virus (HBV)-related chronic liver disease or liver cirrhosis.

Therefore, this study aims to identify HCMV and EBV infection in patients with ACLF-based HBV in our hospital. The specific objective of this study was to explore the HCMV and EBV infection rate, the risk factors of these infections, and their influence on the liver function of patients with ACLF-based HBV, which will improve the survival of these patients through an effective intervention.

## Methods

### Patients

The databases of patients with liver disease were reviewed in our hospital, and patients diagnosed with ACLF due to HBV-related chronic liver disease or liver cirrhosis were identified. HCMV/EBV-infected and –uninfected patients were included in this retrospective study. Eligible patients must have been hospitalized at First Affiliated Hospital, College of Medicine, Zhejiang University from 1 January 2016 to 31 May 2018. The demographics, clinical characteristics, and experimental results, such as liver function and HBV DNA level, were reviewed by a trained team of physicians and entered in duplicate into a computerized system.

### Enrolment criteria

ACLF was defined in accordance with the following criteria specified by the Asian Pacific Association for the Study of the Liver (2014) [13] and the Guideline for Diagnosis and Treatment of Liver Failure in China [21]: (1) acute deterioration of pre-existing HBV-related chronic liver disease or liver cirrhosis; (2) extreme fatigue with severe digestive symptoms, such as observable anorexia, abdominal distension, or nausea and vomiting; (3) progressively worsening jaundice within a short period (serum total bilirubin of ≥10 mg/dL or daily elevation of ≥1 mg/dL); (4) a haemorrhagic tendency, with a prothrombin activity of ≤40% (or international normalized ratio (INR) ≥ 1.5); (5) decompensation ascites; and (6) with or without hepatic encephalopathy. The absence of any of these six criteria precluded a diagnosis of ACLF.

### Exclusion criteria

Patients meeting the following criteria were excluded: (1) ACLF due to HBV-unrelated chronic liver disease or liver cirrhosis, such as alcoholism, autoimmunity, hepatolenticular degeneration, non-alcoholic fatty liver disease, etc.; (2) patients with hepatocellular carcinoma complications; (3) patients aged below 18 years old; (4) women undergoing pregnancy and lactation; (5) patients with AIDS; (6) patients with underlying diseases requiring long-time corticosteroids or immunosuppressive treatments. Patients who met any of these six criteria were excluded from this study.

### Quantitative polymerase chain reaction assay for HCMV and EBV deoxyribonucleic acid (DNA)

HCMV and EBV DNA was extracted from whole-blood samples and detected using a commercial DNA extraction kit (Daan Gene Co. Ltd., Zhongshan University, China) following the manufacturer’s instructions. Quantitative polymerase chain reaction (PCR) was performed using a TaqMan PCR Kit (Daan Gene Co. Ltd., Zhongshan University, China) following the manufacturer’s instructions and run on a real real-time PCR system (Stratagene MX3000P, Agilent Technologies, Santa Clara, CA, USA). Positive and negative controls were included in the quantitative PCR process. The viral loads were denoted in IU/mL. On the basis of the detection limit of the PCR kit, EBV and HCMV DNA loads of <500 copies/mL and ≥ 500 copies/mL were considered negative and positive, respectively. The accuracy was evaluated by an internal quality control, and the coefficient of variation was within an acceptable range.

### Risk factors of HCMV and EBV infection

Previous studies revealed that among organ transplant and HSCT recipients, the risk factors of HCMV and EBV infection were age, male gender, T-cell depletion, immunosuppressive agents, serostatus matching, acute and chronic graft-versus-host disease, rejection and use of human leukocyte antigen (HLA)-mismatched or unrelated donors [3, 5, 6, 11, 12, 22]. However, the potential risk factors of HCMV and EBV infection in immunocompetent and not severely immunocompromised patients, such as those with ACLF due to HBV infection, remain unclear. Previous reports have described the interactions between HBV and HCMV [23, 24], and between HBV and EBV [25]. Thus, we attempted to explore whether in addition to gender and age, HBV-related indexes, including the proliferation state (HBV DNA levels and HBV DNA < 1000 IU/mL), serological status of HBV (HBsAg levels, HBcAb levels, HBeAg positivity (>0.18 PEIU/ml)) and liver cirrhosis are risk factors for HCMV and EBV infection in patients with ACLF due to HBV infection.

### Liver function, child-Pugh score, and MELD score

Liver function, including albumin, alanine aminotransferase (ALT), aspartate aminotransferase (AST), albumin/globulin (A/G), total bilirubin (TB); direct bilirubin (DB), and γ-glutamyl transpeptidase (GGT), was reviewed by Student’s t tests or Mann-Whitney tests. The indexes above that were significantly different were further analysed by binary logistic regression.

The Child-Pugh [26] and model for end-stage liver disease (MELD) [27] scores can be used to accurately assess the liver function and prognosis of patients with end-stage liver disease and liver failure. In addition to the aforementioned functional indexes, we also calculated the Child-Pugh and MELD scores of the patients. The calculation for the MELD score is as follows: MELD score = 3.8 × loge (bilirubin[mg/dL]) + 11.2 × loge(INR) + 9.6 × loge (creatinine [mg/dL]) + 6.4 (aetiology: 0 if cholestatic or alcoholic; otherwise, 1) [27].

### Statistical analysis

Statistical analyses were performed using SPSS software. The results were expressed as the means ± standard deviations, median (quartile) and percentages. The means for continuous variables were compared by using independent-group Student’s t tests for normally distributed data (age, albumin, TB, DB, MELD score) and Mann-Whitney tests for non-normally distributed data (ALT, AST, GGT, Child-Pugh score). The categorical variables were analysed by performing chi-square or Fisher’s exact tests. The risk factors of HCMV and EBV infection as well as the relationship between liver function and HCMV/EBV infection were analysed using binary logistic regression. All *p*-values were based on a two-tailed test of significance.

## Results

### HCMV and EBV infection

**Fig. 1:**
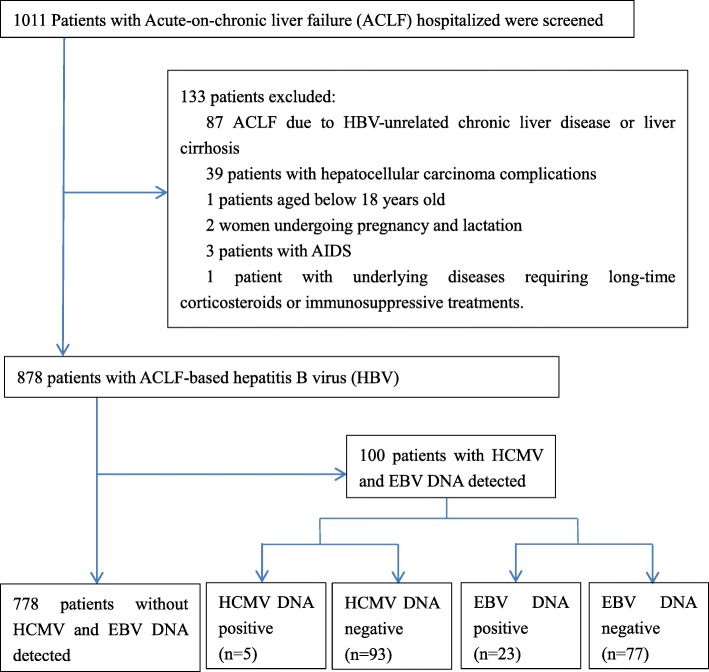
Flow chart of patient selection in the study

As described in Fig. 1 and Table 1, a total of 1011 patients hospitalized in our hospital with ACLF were screened between 1 January 2016 and 1 September 2017. A total of 878 subjects were hospitalized due to ACLF due to HBV, and the others were excluded. A total of 100 patients were assessed for HCMV and EBV, of which five (5.0%) patients were HCMV DNA-positive, with a mean of 1.39 × 104 copies/mL, and 23 (23.0%) patients were EBV DNA-positive, with a mean of 2.7 × 103 copies /mL. In addition, one patient was both HCMV DNA- and EBV DNA-positive. The subjects consisted of 81 men and 19 women, with a mean age of 47.7 years old. A total of 19.0% of the patients were at least 60 years old. The demographic and clinical characteristics of the participants are presented in Table 1.

**Table 1:** Characteristics of patients with acute-on-chronic liver caused by HBV infection

	Total (*n* = 100)	HCMV			EBV		
		Infected (*n* = 5)	Un-infected (*n* = 95)	*P*	Infected (*n* = 23)	Un-infected (*n* = 77)	*P*
Sex (M/F)	81/19	5/0	76/19	0.580	17/6	64/13	0.367
Age^a^	47.68 ± 12.520	45.00 ± 13.191	47.82 ± 12.541	0.626	52.65 ± 15.098	46.19 ± 11.335	0.067
≥ 60 yr	19 (19.0%)	1 (20.0%)	18 (18.9%)	1.000	8 (34.8%)	11 (14.3%)	0.037
Liver Cirrhosis	46 (46.0%)	2 (40.0%)	44 (46.3%)	1.000	12 (52.2%)	34 (44.2%)	0.498
Underling Conditions^b^							
Hypertension	13 (13.0%)	1 (20.0%)	12 (12.6%)	0.509	2 (8.7%)	11 (14.3%)	0.727
Diabetes Mellitus	11 (11.0%)	1 (20.0%)	10 (10.5%)	0.449	4 (17.4%)	7 (9.1%)	0.271
Coronary Atherosclerotic Heart Disease	4 (4.0%)	0 (0.0%)	4 (4.2%)	1.000	1 (4.3%)	3 (3.9%)	1.000
Chronic Bronchitis and Pulmonary emphysema	1 (1.0%)	0 (0.0%)	1 (1.1%)	1.000	0 (0.0%)	1 (1.3%)	1.000
Ulcerative Colitis	1 (1.0%)	1 (20.0%)	0 (0.0%)	0.086	0 (0.0%)	1 (1.3%)	1.000
Schizophrenia	2 (2.0%)	0 (0.0%)	2 (2.1%)	1.000	0 (0.0%)	2 (2.6%)	1.000
Chronic kidney disease	1 (1.0%)	0 (0.0%)	1 (1.1%)	1.000	0 (0.0%)	1 (1.3%)	1.000
Neoplasm							
Gastric Carcinoma	1 (1.0%)	0 (0.0%)	1 (1.1%)	1.000	0 (0.0%)	1 (2.2%)	1.000
Renal Carcinoma	1 (1.0%)	0 (0.0%)	1 (1.1%)	1.000	1 (7.7%)	0 (0.0%)	0.224

Note: ^a^mean ± standard deviation, years; ^b^one patient underling conditions included coronary atherosclerotic heart disease, hypertension, chronic bronchitis and emphysema pulmonum; one patient coexisting conditions included diabetes Mellitus, hypertension; and one patient coexisting conditions included coronary atherosclerotic heart Disease, diabetes mellitus, hypertension

### Risk factors for HCMV and EBV infection

Among the aforementioned possible risk factors, we found that HBV DNA < 1000 IU/mL was a risk factor for HCMV infection (*P* = 0.003), with a dramatically increased risk of 34.00-fold (95% CI: 3.453–334.800). The ages of the EBV-infected patients were older than the uninfected patients. A greater number of EBV-infected patients were > 60 years of age. Age, especially older than 60 years (*P* = 0.034, *P* = 0.033) was a risk factors for EBV infection by 1.042-fold and 3.200-fold, respectively (95% CI: 1.003–1.082, 1.098–9.324). (Table 1, Table 2).

**Table 2:** Binary logistic analysis for HCMV, EBV risk factors

Variable	HCMV			EBV		
	OR	95% CI	*P*	OR	95% CI	*P*
Age	0.981	0.909–1.059	0.623	1.042	1.003–1.082	0.034
Age ≥ 60 yr	1.069	0.113–10.153	0.953	3.200	1.098–9.324	0.033
Male	0.000	0.000–0.000	0.998	1.738	0.575–5.248	0.327
Liver Cirrhosis	0.773	0.123–4.837	0.783	1.380	0.542–3.510	0.499
HBV DNA levels	1.000	0.999–1.000	0.241	1.000	1.000–1.000	0.890
HBV DNA (<1000 IU/ml)	34.000	3.453–334.800	0.003	0.900	0.228–3.546	0.880
HBsAg titer	1.000	1.000–1.000	0.607	1.000	1.000–1.000	0.317
HBcAb titer	1.108	0.859–1.430	0.429	0.956	0.769–1.198	0.687
HBeAg positive (>0.18PEIU/ml)	0.773	0.123–4.837	0.783	2.187	0.844–5.669	0.107

Note: *HCMV* human cytomegalovirus, *EBV* Epstein–Barr virus

### Liver function, child-Pugh score, and MELD score

All indexes of liver function, Child-Pugh score, and MELD score were analysed between infected and uninfected patients. Only the ALT and AST levels in HCMV-uninfected patients were significantly lower than in HCMV-infected patients (*P* = 0.001, *P* = 0.002). When the above indexes (ALT and AST) were further analysed using binary logistic regression, we only found that there was relationship between lower ALT levels and HCMV infection (*P* = 0.039, OR 1.067). However, the albumin level was lower in EBV-infected patients (29.72 ± 3.626 g/L) than in uninfected patients (33.19 ± 4.217 g/L). This difference was statistically significant (*P* = 0.001). EBV-infected patients achieved significantly higher Child-Pugh scores (12.0 (11.0, 13.0) VS 10.0 (9.0, 12.0), *P* = 0.008). When we further analysed the relationship between the albumin level, Child-Pugh score and EBV infection using binary logistic regression, we found that EBV-infected patients had lower albumin levels and higher Child-Pugh scores (P = 0.001, OR 1.242; *P* = 0.009, OR 1.493). (Tables 3, 4 and 5).

**Table 3:** Liver function between HCMV infected and un-infected patients

	HCMV infected (*n* = 5)	HCMV un-infected (*n* = 95)	*P*
ALT (U/L)	34.0 (30.0, 48.0)	166.0 (87.0, 446.0)	0.001
AST (U/L)	45.0 (43.5, 52.0)	134.0 (78.0, 254.0)	0.002
Albumin (g/L)	31.86 ± 3.002	32.42 ± 4.397	0.779
TB (U/L)	358.20 ± 191.525	328.91 ± 119.722	0.606
DB (U/L)	241.80 ± 125.398	228.74 ± 85.521	0.746
GGT (U/L)	46.0 (38.5, 142.5)	75.0 (55.0, 125.0)	0.300
Child-pugh Score	12.0 (9.0, 12.0)	11.0 (10.0, 12.0)	0.994
Meld Score	20.43 ± 2.328	23.54 ± 5.759	0.235

Note: *ALT* alanine aminotransferase, *AST* aspartate aminotransferase, *A/G* Albumin/Globulin, *TB* total bilirubin, *DB* direct bilirubin, *GGT* γ-glutamyl transpeptadase

**Table 4:** Liver function between EBV infected and un-infected patients

	EBV infected (*n* = 23)	EBV un-infected (*n* = 77)	*P*
ALT (U/L)	162.0 (76.0, 504.0)	153.0 (74.5.0, 408.0)	0.734
AST (U/L)	133.0 (50.0, 332.0)	130.0 (69.0, 241.0)	0.889
Albumin (g/L)	29.72 ± 3.626	33.19 ± 4.217	0.001
TB (U/L)	291.52 ± 109.996	341.98 ± 124.966	0.084
DB (U/L)	202.10 ± 74.653	237.55 ± 89.320	0.087
GGT (U/L)	70.0 (46.0, 92.0)	78.0 (54.5, 128.0)	0.206
Child-pugh Score	12.0 (11.0, 13.0)	10.0 (9.0, 12.0)	0.008
Meld Score	23.48 ± 4.551	23.35 ± 5.992	0.925

Note: *ALT* alanine aminotransferase, *AST* aspartate aminotransferase, *A/G* Albumin/Globulin, *TB* total bilirubin, *DB* direct bilirubin, *GGT* γ-glutamyl transpeptadase

**Table 5:** Binary logistic analysis for liver function and HCMV, EBV infection

Virus	variable	OR (95% CI)	*P*
HCMV	ALT	1.067 (1.003~ 1.135)	0.039
	AST	1.078 (0.994~ 1.169)	0.071
EBV	Albumin	1.242 (1.088~ 1.416)	0.001
	Child-pugh Score	1.493 (1.106~ 2.015)	0.009

Note: *HCMV* human cytomegalovirus, *EBV* Epstein–Barr virus

## Discussion

Human herpes viruses include herpes simplex virus type 1 (HSV-1), herpes simplex virus type 2 (HSV-2), varicella zoster virus (VZV), HCMV, EBV, human herpesvirus 6 (HHV-6), human herpesvirus 7 (HHV-7), and human herpesvirus 8 (HHV-8). Although HCMV and EBV are more studied in this respect, after initial infections with no or mild symptoms, all human herpes viruses may become latent under certain conditions. However, most studies of latent HCMV and EBV infection have focused on immunosuppressed individuals. In this study, we aimed to focus our investigation on HCMV and EBV in immunocompetent individuals.

Our study evaluates a cohort of 100 hospitalized patients with ACLF due to HBV infection, of which 5.0% patients presented with HCMV DNA, 23.0% patients presented with EBV DNA, and one patient was positive for both HCMV DNA and EBV DNA. HBV DNA < 1000 IU/mL was a risk factor for HCMV infection, and age, especially older than 60 years, were risk factors for EBV infection. HCMV-infected patients had lower ALT levels, indicating that HCMV infection may not increase liver injury. In contrast, EBV infection possibly influenced liver function, as EBV-infected patients had lower albumin levels and higher Child-Pugh scores.

By reviewing the medical records and laboratory data, we found that of the 69 patients in this study in whom HCMV IgG and EB viral capsid antigen (EB-VCA) IgG were measured, all were HCMV IgG- and EB-VCA IgG-positive. HCMV and EBV primary infection mostly occur in childhood. Thus, in this study, it is likely that the observed HCMV and EBV infections were due to reactivation rather than primary infection.

Previous studies have investigated the association between HCMV infection and HBV infection [23, 28–30]. In the present study, we found that 5.0% of patients were co-infected with HCMV, and this finding was similar to that reported by Lian et al. [30]. Limited studies have explored the association between EBV infection and HBV infection [25]. An et al. [25] reported that compared with HBV infection (9.10%), EBV infection occurred more frequently in patients with HBV-related liver cirrhosis (40.0%) and liver carcinoma patients (25.0%). In the present study, we found that 23.0% of the patients were infected with EBV, which differed from the previously reported infection rate because chronic HBV infection and liver cirrhosis were both included in our study.

Immunosuppressive drugs, high-dose corticosteroids, T-cell depletion, acute and chronic GVHD, rejection, and virus coinfection were identified as the most common risk factors for HCMV or EBV infection [3, 5, 6, 11, 12, 22]. These risk factors were determined mainly based on immunodeficient or immunosuppressed patients, such as organ transplant recipients, HSCT recipients, and patients with AIDS. Few studies have considered immunocompetent and not severely immunocompromised patients. In the present study, we focused on patients with ACLF due to HBV infection.

ACLF is defined as an acute hepatic insult manifesting as jaundice and coagulopathy that is complicated within 4 weeks by ascites and/or encephalopathy in patients with previously diagnosed or undiagnosed chronic liver disease [13, 21] with various degrees of immune disorders. Considering the relationship among HCMV, EBV, and HBV [23, 25, 28–30], we attempted to explore whether HBV-related indexes and liver cirrhosis are potential risk factors in addition to age and gender.

We found that HCMV-infected patients had lower HBV DNA levels than HCMV-uninfected patients (*P* = 0.001). Eighty percent of the HCMV-infected patients had HBV DNA < 1000 IU/mL, which was greater than the number of HCMV-uninfected patients with similar levels (9.5%). In addition, we found that HBV DNA < 1000 IU/mL was a risk factor for HCMV infection. HBV was strongly correlated with HCMV. Our findings were similar to those of previous reports [23, 24]. Cavanaugh, V. J. et al. [24] discovered that cytokines produced by murine cytomegalovirus inhibited HBV replication and gene expression. Bayram, A. et al. [23] revealed that the inflammation caused by HCMV can contribute to viral clearance during chronic HBV infection. Therefore, we suggest that HBV and HCMV replication may mutually inhibit each other. Thus, when HBV replication is inactive, as indicated by a lower titre of HBV DNA, especially HBV DNA < 1000 IU/mL, HCMV replication activity is facilitated. However, the specific mechanism underlying this phenomenon must be further studied.

Our previous study posited that male donors, conditioning regimens including ATG and GVHD prophylaxis, and prednisone increase the risk of EBV infection in patients receiving HSCT [5]. However, EBV infection and its risk factors were rare in patients who are not severely immunocompromised, such as those with ACLF. In the present study, HBV-related indexes, including proliferation state, serological status of HBV and liver cirrhosis, were not risk factors of EBV infection in HBV-related ACLF. However, age was a risk factor for EBV infection, especially in patients > 60 years of age. XM. Zhang [31] previously reported that the rate of EBV-1 infection in this age group was significantly higher than that in the < 40 age group. Age was related to EBV-1 infection in patients with chronic periodontitis. It was suggested that the immune system and the environment of the host change with the age, and thus, latent EBV was released and propagated in the host cell [31].

Bayram, A. et al. [23] indicated that the mean ALT and intrahepatic HBV DNA levels in patients with HCMV co-infected with chronic viral hepatitis B were lower than those in patients without HCMV co-infection. Similarly, we found that the levels of ALT, AST, and HBV DNA were lower in patients with ACLF due to HBV co-infection with HCMV. However, further analysis using binary logistic regression indicated that only the ALT level was related to HCMV infection. Therefore, we suggest that HCMV infection may inhibit HBV proliferation, decrease inflammatory activity, reduce liver cell damage, and reduce ALT levels. However, the specific mechanism underlying this phenomenon must be further studied.

Previous studies have reported that EBV infection causes acute liver failure [15–17]. In the present study, EBV-infected patients had lower albumin levels than uninfected patients. In addition, EBV-infected patients also had higher Child-Pugh scores. Thus, EBV infection may be related to lower albumin and higher Child-Pugh score. EBV infection may increase liver injury and decrease the liver synthesis ability in ACLF patients. Furthermore, Child-Pugh score is a suitable prognostic evaluation indictor for patients with end-stage liver disease; a higher score corresponds to higher mortality in the short term [26]. Thus, the influence of EBV infection on liver function may result in poor prognosis.

This work is limited by a number of factors. First, a slight bias is present in any retrospective study, which will result in a certain statistical bias. Second, we did not conduct follow-up for the included patients and did not analyse the confirmed influence of prognosis on HCMV and EBV infection in the short and long terms. Third, we unfortunately did not include all human herpes viruses in this study. Fourth, we found that HCMV-infected patients had lower HBV DNA levels, and thus HCMV viral replication is likely to be detrimental to HBV replication in these 5 HCMV-infected cases. This may be due to the inflammatory cytokines caused by HCMV contributing to viral clearance during chronic HBV infection. However, we unfortunately did not measure the proinflammatory cytokines released by HCMV that prevent HBV replication. Fifth, we cannot distinguish between primary infection and reactivation of HCMV and EBV infection for all patients, although there all 69 patients in our study who were evaluated were found to be HCMV IgG- and EBV IgG-positive. We will address these drawbacks in our future studies. Finally, although we found that EBV-infected patients had lower albumin levels and higher Child-Pugh scores, as indicated by binary logistic regression, this may be associated with overall physiological decline in these patients. Although this study does have limitations, our evaluation of patients with HBV-related ACLF may begin to fill in gaps of knowledge on HCMV and EBV infection in immunocompetent patients.

## Conclusions

This study revealed that HCMV and, more notably, Epstein-Barr virus (EBV) were detected in patients with ACLF due to HBV infection. Lower replication levels of HBV (HBV DNA < 1000 IU/mL) was associated with an increased probability of HCMV infection; age, especially older than 60 years, were associated with EBV infection. HCMV infection may inhibit HBV proliferation and reduce liver injury, while EBV infection possibly influenced liver function, as EBV-infected patients had lower albumin levels and higher Child-Pugh scores. HCMV co-infection did not increase liver injury. However, co-infection with EBV may influence liver function and may induce poor prognosis in HBV-related ACLF. Thus, attention should be paid to the possibility that older patients, especially those older than 60 years with ACLF due to HBV, may be infected with EBV. In this case, effective antiviral therapy may be needed; otherwise, EBV infection may influence the patients^,^prognosis.

## Abbreviations

A/G: Albumin/Globulin
ACLF: Acute-on-chronic liver failure
AIDS: immune deficiency syndrome
ALF: Acute liver failure
ALT: Alanine aminotransferase
AST: Aspartate aminotransferase
DB: Direct bilirubin
DNA: Deoxyribonucleic acid
EBV: Epstein-Barr virus
GGT: γ-glutamyl transpeptadase
HBeAg: Hepatitis B e antigen
HBsAg: Hepatitis B surface antigen
HBV: Hepatitis B virus
HCMV: Human cytomegalovirus
HLA: Human leukocyte antigen
HSCT: Hematopoietic stem cell transplant recipients
INR: International normalized ratio
MELD: Model for end-stage liver disease scores
PCR: Polymerase chain reaction
TB: Total bilirubin
